# Lymphoplasmacytic Gastritis in Cheetahs Under Human Care: A Bile Acid-Driven Gastroenteropathy Arising from Disrupted Feeding Ecology

**DOI:** 10.3390/ani16101494

**Published:** 2026-05-13

**Authors:** Adrian S. W. Tordiffe

**Affiliations:** 1 Greens Zoological Rescue and Rehabilitation Centre, Jamnagar 361142, Gujarat, India; adrian.tordiffe@up.ac.za; 2 Department of Paraclinical Sciences, Faculty of Veterinary Science, University of Pretoria, Onderstepoort 0110, South Africa

**Keywords:** *Acinonyx jubatus*, bile acid signaling, digestive physiology, feast-fast feeding ecology, fat maldigestion, carnivore nutrition, carnivore gastrointestinal disease

## Abstract

Lymphoplasmacytic gastritis (LPG) is one of the most prevalent chronic diseases affecting cheetahs (*Acinonyx jubatus*) under human care, yet its underlying cause remains unresolved. Gastric inflammation occurs in the majority of adult captive cheetahs but is uncommon in free-ranging populations, suggesting that management-related factors contribute to disease pathogenesis. This review proposes that LPG represents a bile acid-driven gastroenteropathy arising from disruption of the natural feeding ecology of the cheetah. In free-ranging systems, cheetahs consume large episodic meals separated by prolonged fasting intervals and ingest whole-prey containing substantial connective tissue and collagen. In captivity, feeding patterns are typically characterized by smaller, more frequent meals and diets dominated by lean skeletal muscle with reduced structural complexity. I hypothesize that this mismatch alters gastric emptying kinetics, disrupts coordinated pancreatic and biliary secretion, and destabilizes fat digestion. Inefficient lipolysis may impair micelle formation and promote bile acid mislocalization within the gastrointestinal tract, increasing mucosal exposure to hydrophobic bile acids capable of inducing chemical epithelial injury. Within this framework, lymphoplasmacytic gastritis is interpreted as a secondary inflammatory reaction to chronic bile acid-mediated mucosal stress rather than a primary immune-mediated disorder. The model also provides a mechanistic explanation for the frequent coexistence of gastritis with fat and protein maldigestion in captive cheetahs. Differential responses to antimicrobial therapy, glucocorticoids, sulfasalazine, pancreatic enzyme supplementation, and bile acid-modifying agents are broadly consistent with this proposed mechanism. Recognition of LPG as a physiologically driven gastroenteropathy has important implications for management, emphasizing restoration of feast–fast feeding patterns, inclusion of collagen-rich carcass components, and targeted modulation of bile acid composition and signaling.

## Simple Summary

Chronic stomach inflammation is one of the most common diseases affecting cheetahs living under human care, yet the underlying cause remains poorly understood. This article proposes that the disease may develop because captive feeding practices differ substantially from the natural feeding behavior of wild cheetahs. Wild cheetahs consume large whole-prey meals separated by prolonged fasting periods, whereas captive cheetahs are often fed smaller meals more frequently and may receive diets low in connective tissue and collagen. The article suggests that these differences may disrupt normal digestion and bile acid movement within the gastrointestinal tract, leading to chemical irritation of the stomach lining and chronic inflammation. This framework may help explain why digestive problems frequently occur alongside gastritis in captive cheetahs and why many current treatments provide inconsistent results. If correct, the hypothesis could improve long-term management by encouraging feeding practices and diet structures that more closely resemble the natural biology of the species.

## 1. Lymphoplasmacytic Gastritis in Cheetahs Under Human Care: An Unresolved Disease with Persistent Pathogenic Assumptions

Lymphoplasmacytic gastritis (LPG) is one of the most prevalent and clinically significant chronic diseases affecting cheetahs (*Acinonyx jubatus*) under human care. Post-mortem surveys across zoological and breeding institutions consistently report gastric inflammatory lesions in most adult captive cheetahs, often irrespective of clinical history [1,2,3,4]. In contrast, free-ranging cheetahs—despite lifelong exposure to infectious agents, dietary variability, and environmental stressors—rarely develop clinically significant gastritis, and when present, lesions are typically mild and incidental [1,2]. In captive populations, LPG is strongly associated with chronic intermittent regurgitation, diarrhea, poor coat quality, weight loss, and declining body condition, contributing substantially to morbidity and premature mortality [2,3,4,5]. These observations suggest that LPG is not an incidental pathological finding, but a central disease process affecting the long-term health of captive cheetahs.

Considerable effort has been directed toward identifying an infectious etiology for LPG, particularly involving *Helicobacter* spp. Multiple *Helicobacter* species have been isolated from the gastric mucosa of both captive and free-ranging cheetahs, yet no consistent association between specific strains and disease severity has been demonstrated [1,3,6]. Importantly, *Helicobacter* organisms are frequently present in clinically normal free-ranging animals, while antimicrobial therapy typically results in only transient clinical or histological improvement, without durable disease resolution [1,2,3]. Furthermore, many clinical cases are treated empirically or based on histopathology alone, without bacterial culture or antimicrobial susceptibility testing. Collectively, these findings suggest that *Helicobacter* infection is unlikely to represent a sufficient primary cause of disease and may instead function as a secondary colonizer or disease modifier within an already compromised gastric environment.

Over the past three decades, alternative explanations for LPG have included chronic stress, genetic predisposition, immune dysregulation, and husbandry-associated factors [2,3,4]. Although each of these may contribute to disease susceptibility, none has provided a unifying mechanistic framework capable of explaining the high prevalence of gastritis in captive cheetahs, and its relative rarity in free-ranging populations. Nutritional factors are frequently acknowledged, yet discussion has largely focused on micronutrients or individual dietary ingredients. By contrast, feeding ecology—including feeding frequency, meal structure, and the physical composition of the diet—has received comparatively little mechanistic attention despite marked divergence from the natural feeding patterns of wild cheetahs.

This review proposes that lymphoplasmacytic gastritis in captive cheetahs is primarily driven by a mismatch between natural feeding ecology and captive feeding practices, with particular emphasis on increased feeding frequency and diets deficient in collagen-rich tissues. I argue that these factors disrupt gastric physiology, digestive coordination, and bile acid handling, ultimately promoting chronic bile acid-mediated chemical injury of the gastric mucosa and secondary immune activation. Within this framework, LPG is interpreted not as a primary immune-mediated disease, but as a histological reaction pattern arising from sustained physiological mismatch between evolved digestive biology and captive management practices.

## 2. Feeding Ecology of the Cheetah: Physiology Shaped by Feast–Fast Cycles

The cheetah is an obligate carnivore whose gastrointestinal physiology evolved under conditions of pronounced nutritional intermittency. In free-ranging systems, prey capture is episodic, meals are large relative to body size, and extended fasting intervals are the norm [7]. This feast–fast pattern imposes strong physiological demands on gastric storage capacity, acid secretion dynamics, pancreatic enzyme release, gallbladder contraction, and enterohepatic bile acid cycling. Rather than functioning independently, these processes operate as coordinated digestive and signaling events that evolved within the context of intermittent feeding.

Under captive management, this feeding ecology is fundamentally altered. To facilitate husbandry, training, and behavioral management, cheetahs are commonly fed smaller meals more frequently, often with supplemental food items provided on intended fasting days. Although total weekly caloric intake may approximate that of free-ranging animals, the temporal structure of intake differs markedly. Frequent feeding reduces the duration of the interdigestive state and may disrupt the cyclical gastrointestinal physiology associated with prolonged fasting.

From a gastric perspective, repeated small meals maintain the stomach in a semi-postprandial state characterized by persistent acid secretion, incomplete gastric emptying, and reduced opportunity for restoration of the mucus–bicarbonate barrier. The absence of prolonged fasting intervals may also limit periods of epithelial recovery and physiological resetting of gastrointestinal motility. Importantly, opportunistic gastric fluid pH measurements obtained during gastroscopy in sedated cheetahs commonly demonstrate strongly acidic gastric contents, often around pH 2, although considerable variability occurs. Subjectively, no obvious correlation has been observed between isolated gastric pH measurements and the apparent severity of gastritis during endoscopic examination. These observations suggest that the proposed mechanism is unlikely to reflect simple hyperacidity alone and support consideration of altered bile acid handling and digestive coordination as potentially more important contributors to mucosal injury.

In addition to altering gastric emptying and biliary signaling, frequent feeding may disrupt the normal interdigestive migrating motor complex (MMC). The MMC is a cyclical fasting-associated pattern of gastrointestinal motility that functions to clear residual luminal contents, regulate microbial populations, and coordinate pancreatic and biliary secretory activity [8,9,10]. Phase III MMC activity is associated with pulsatile pancreatic enzyme secretion and gallbladder contraction, contributing to the orderly progression and recycling of bile acids through the small intestine [8,9]. Repeated ingestion of small meals suppresses MMC activity and interrupts the normal interdigestive cycle of coordinated gastrointestinal motility [9,10]. Although direct studies of MMC physiology in cheetahs are lacking, disruption of fasting-associated motility may be particularly relevant in obligate carnivores adapted to prolonged fasting intervals. Loss of coordinated interdigestive motility could contribute to impaired bile acid clearance, altered duodenogastric pressure gradients, and instability of pancreatic–biliary synchronization.

Feeding frequency also has important implications for biliary physiology. Gallbladder contraction and bile acid delivery are optimally stimulated by discrete meals containing sufficient fat to trigger coordinated cholecystokinin release. Frequent small meals may induce repeated submaximal gallbladder contractions, resulting in inefficient bile delivery and poor synchronization with pancreatic lipase activity. These conditions predispose to functional fat maldigestion despite preserved pancreatic exocrine capacity.

Experimental manipulation of feeding frequency in captive cheetahs provides indirect support for the physiological relevance of feeding ecology. Reducing feeding frequency while maintaining total weekly caloric intake has been shown to improve fecal consistency and locomotor behavior without evidence of sustained physiological stress following an initial adaptation period [11]. These findings suggest that frequent feeding is not inherently protective and may compromise gastrointestinal stability.

Taken together, deviation from natural feeding ecology in cheetahs should be regarded as a potentially important physiological perturbation with direct relevance to gastric, pancreatic, and biliary function, rather than as a benign husbandry modification.

## 3. Dietary Structure, Collagen Deficiency, and Functional Digestive Failure

In addition to feeding frequency, the structural composition of diets provided to captive cheetahs differs markedly from that of natural prey. Free-ranging cheetahs consume whole carcasses that include skin, fascia, cartilage, tendons, bone fragments, hair and viscera, whereas diets provided in captivity are frequently dominated by lean skeletal muscle or mechanically processed meat with substantially lower connective tissue complexity [12,13]. This shift substantially alters the physical and biochemical properties of the ingested meal.

The structural properties of whole-prey may also contribute to digestive stability in strict carnivores. Depauw et al. proposed the concept of “animal fiber,” referring to the relatively indigestible connective tissue, cartilage, bone fragments, and hair naturally present in prey but largely absent from processed meat diets fed to many captive carnivores [13]. These structural components may function analogously to insoluble fiber in omnivores by influencing gastric retention, intestinal transit, microbial fermentation, and fecal consistency [13,14]. Experimental inclusion of connective tissue fractions in cheetah diets altered digestive parameters and stool quality, supporting the concept that prey structure contributes directly to gastrointestinal physiology rather than simply nutrient provision [13].

Collagen-rich tissues should therefore be considered a functional structural component of the diet, rather than merely a protein source. Compared with lean skeletal muscle, collagen-rich tissues are digested more slowly and may influence gastric viscosity, gastric emptying kinetics, and the temporal coordination of nutrient delivery to the proximal small intestine [15]. These effects could prolong synchronized exposure of dietary lipids to pancreatic lipase and bile acids during digestion. In contrast, low-collagen diets may favor relatively rapid gastric emptying and premature nutrient delivery to more distal intestinal segments, potentially destabilizing lipolysis and micelle formation despite apparently adequate pancreatic enzyme secretion.

Clinical response patterns in affected cheetahs support this interpretation. Supplementation with proteolytic enzymes alone, including plant-derived proteases such as bromelain or papain, often fails to stabilize fecal quality or gastrointestinal function. In contrast, supplementation with pancreatic enzyme preparations containing lipase often results in rapid improvement in stool consistency and nutrient utilization. This response pattern argues against primary pancreatic exocrine insufficiency and instead supports the possibility of functionally impaired digestive coordination driven by diet structure and feeding pattern.

Glycine availability may further influence susceptibility to digestive instability. Glycine plays a central role in collagen turnover, antioxidant defense, glutathione synthesis and conjugation of endogenous and microbial metabolites. Metabolomic studies in captive cheetahs suggest relatively constrained glycine availability and demonstrate that glycine supplementation alters metabolic and fermentation profiles [12]. Diets chronically low in collagen-rich tissues may therefore reduce metabolic buffering capacity while simultaneously altering digestive dynamics, potentially increasing vulnerability to bile acid-mediated epithelial stress.

Taken together, frequent feeding of low-collagen, muscle-meat-dominant diets may create a state of functional digestive failure in susceptible cheetahs. Within this framework, pancreatic enzyme secretion may be quantitatively adequate yet kinetically ineffective, bile acids may be produced normally yet inadequately coordinated with digestion, and fat digestion becomes unstable and inconsistent.

## 4. From Fat Maldigestion to Bile Acid Mislocalization

Effective fat digestion depends on precise coordination between gastric emptying, pancreatic lipase secretion, bile acid delivery, and intestinal motility. Efficient micelle formation maintains bile acids in solution and facilitates their orderly progression through the small intestine to the terminal ileum, where active reabsorption occurs [15,16]. When lipolysis is incomplete, bile acids may remain poorly incorporated into micelles, potentially increasing the proportion of unbound bile acids within the intestinal lumen.

Bile acids are not a homogeneous class of molecules, and their biological effects are determined not only by concentration but also by composition and localization. Hydrophobic bile acids exert potent detergent effects on epithelial membranes and may promote mucosal inflammation, whereas more hydrophilic bile acids contribute to epithelial protection and mucosal homeostasis [16,17,18]. In addition to their physicochemical properties, bile acids function as endocrine-like signaling molecules through receptors such as the farnesoid X receptor (FXR) and Takeda G-protein-coupled receptor 5 (TGR5), which regulate epithelial integrity, immune tolerance, motility, and metabolic homeostasis [16,18]. Disruption of bile acid composition, localization, or receptor activation therefore has consequences that extend well beyond lipid emulsification.

It is also important to distinguish bile acid-mediated injury from simple hyperacidity. Duodenal contents entering the proximal small intestine are normally alkaline overall because of biliary and pancreatic secretions; however, refluxed bile acids and bile salts, particularly more hydrophobic species, retain membrane-disruptive and pro-inflammatory properties capable of inducing epithelial injury independent of luminal acidity [19,20]. Opportunistic spot measurements of gastric pH obtained during gastroscopy in sedated cheetahs have not subjectively shown any obvious relationship with the apparent severity of gastritis, suggesting that low gastric pH alone is unlikely to represent the principal driver of disease.

Disruption of micellar stability has direct consequences for enterohepatic circulation. Reduced ileal reclamation of bile acids could increase compensatory hepatic bile acid synthesis and alter bile acid pool composition [16,21]. At the same time, impaired synchronization between gastric emptying and duodenal bile delivery may alter pressure gradients across the pylorus, increasing the likelihood of retrograde movement of bile-containing duodenal contents into the stomach.

When mislocalized, bile acids gain access to the gastric lumen, where they act as potent chemical stressors to the mucosa. Unlike acid-mediated injury, which is partially mitigated by mucus and bicarbonate secretion, bile acid-mediated chemical injury disrupts epithelial membranes, increases mucosal permeability, impairs tight junction integrity, and promotes inflammatory signaling [19,22]. Repeated exposure can result in chronic gastric injury even in the absence of hyperacidity, infection, or overt ulceration.

In addition to their detergent properties, bile acids function as key signaling molecules regulating epithelial integrity, immune tolerance, microbial composition and gastrointestinal motility. Alterations in bile acid composition and distribution influence microbial generation of secondary bile acids and impair activation of bile acid receptors such as FXR and TGR5 [16,18]. Dysregulation of these pathways has been implicated in chemically driven enteropathies in domestic species [16,18] and provides a mechanistic framework for understanding persistent mucosal inflammation in captive cheetahs.

**Figure 1:**
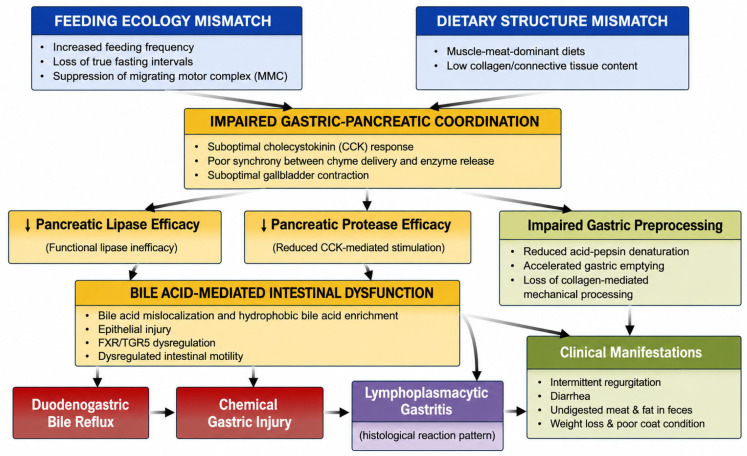
Proposed pathogenesis of lymphoplasmacytic gastritis in cheetahs under human care. Disruption of natural feeding ecology through increased feeding frequency and diets deficient in collagen-rich tissues alters gastric emptying kinetics and reduces coordinated cholecystokinin-mediated pancreatic enzyme release. These changes destabilize fat digestion and micelle formation, resulting in bile acid mislocalization within the gastrointestinal tract. Exposure of gastric and intestinal mucosa to hydrophobic bile acids promotes epithelial injury, inflammatory signaling, and lymphoplasmacytic infiltration. Parallel disruption of gastric–pancreatic coordination contributes to functional fat and protein maldigestion. Interdigestive processes such as the migrating motor complex may further contribute to bile acid mislocalization when feeding frequency is increased. Together, these processes generate the clinical syndrome characterized by gastritis, diarrhea, and poor nutritional status observed in captive cheetahs.

Within this framework, bile acid mislocalization provides a direct mechanistic link between functional fat maldigestion and chronic gastric and intestinal disease. Chemical injury, rather than infection or primary immune dysregulation, becomes the dominant driver of mucosal pathology, setting the stage for the lymphoplasmacytic inflammatory response characteristic of gastritis in captive cheetahs (Figure 1).

## 5. Chemical Gastritis, Lymphoplasmacytic Infiltration, and Secondary Immune Activation

Chronic exposure of the gastric mucosa to bile acids represents a form of chemical injury fundamentally distinct from acid-mediated or infectious gastritis. While the stomach is well adapted to tolerate low luminal pH and pepsin activity, it is comparatively poorly protected against bile acids, particularly hydrophobic species that disrupt epithelial membranes and compromise tight junction integrity [19,20,22]. Recurrent duodenogastric reflux may therefore constitute a persistent injurious stimulus, even in the absence of hyperacidity or overt ulceration.

At the histological level, bile acid-mediated epithelial injury increases mucosal permeability, permitting luminal antigens and endogenous damage-associated molecular patterns to access the lamina propria. This may result in sustained immune stimulation characterized predominantly by lymphoplasmacytic infiltration. Importantly, this inflammatory pattern is non-specific and should be interpreted cautiously as a reaction phenotype rather than direct evidence of a particular initiating cause. Similar lymphoplasmacytic responses occur throughout the gastrointestinal tract in a wide range of chemically or metabolically driven enteropathies in domestic species.

Within the framework proposed here, lymphoplasmacytic gastritis in captive cheetahs is interpreted as a downstream consequence of chronic epithelial stress rather than a primary immune-mediated disorder. This interpretation may explain several otherwise paradoxical observations. Histological severity often correlates poorly with clinical signs, consistent with chronic adaptive inflammation rather than episodic immune-mediated disease [1,2,3,4]. Antimicrobial therapy directed at *Helicobacter* spp. may transiently reduce inflammatory burden, possibly by decreasing bacterial amplification of mucosal injury, yet fails to produce durable disease resolution [1,3]. Likewise, immunosuppressive therapies frequently provide limited or inconsistent benefit.

Beyond their detergent properties, bile acids function as potent signaling molecules capable of modulating inflammatory pathways. Exposure of gastric and intestinal epithelium to bile acids may activate nuclear factor-κB (NF-κB) signaling pathways, increase pro-inflammatory cytokine production, and promote recruitment of lymphocytes and plasma cells [22,23]. Concurrently, dysregulated bile acid composition and localization may impair activation of bile acid receptors, including the FXR and TGR5, both of which play central roles in maintaining epithelial integrity, immune tolerance, and coordinated gastrointestinal motility [16,18]. Loss of these regulatory signals could lower the threshold for chronic mucosal inflammation and favor persistence of epithelial injury.

Once established, gastric inflammation may further impair antral motility and pyloric coordination, increasing the likelihood of continued bile reflux and reinforcing the cycle of chemical injury. In this manner, gastritis becomes self-sustaining, not because it is primary, but because the underlying physiological disturbance remains unresolved.

### 5.1. Resolving the Association Between Gastritis and Maldigestion

A long-standing and unresolved feature of gastrointestinal disease in captive cheetahs has been the frequent coexistence of lymphoplasmacytic gastritis with clinical and biochemical evidence of maldigestion and malabsorption [1,2,3,4]. Under prevailing disease models, this association has remained difficult to explain because primary gastric inflammation alone offers no clear mechanism for impaired pancreatic enzyme efficacy or nutrient assimilation.

Within the present framework, this apparent paradox may be more readily resolved. Functional impairment of fat digestion and bile acid handling represents an upstream disturbance from which both gastritis and maldigestion arise as parallel consequences. Ineffective lipolysis and unstable micelle formation may promote bile acid mislocalization while simultaneously impairing digestive efficiency within the small intestine. Gastritis and maldigestion are therefore interpreted not as directly causally related phenomena but rather co-generated manifestations of the same physiological disturbance.

This interpretation aligns with several clinical observations. The severity of gastric inflammation does not consistently predict the degree of maldigestion, and therapeutic interventions often improve one domain without fully resolving the other. By contrast, correction of feeding structure and digestive coordination often improves both concurrently, consistent with a shared upstream mechanism.

### 5.2. Functional Protein Maldigestion from Disrupted Gastric–Pancreatic Coordination

In a subset of affected cheetahs, fecal examination reveals intact or poorly digested muscle fibers, indicating true protein maldigestion rather than isolated malabsorption. This finding may reflect disruption of coordinated gastric and pancreatic digestive function rather than primary pancreatic protease deficiency.

Low-collagen, muscle-meat-dominant diets combined with altered feeding frequency may result in suboptimal cholecystokinin-mediated pancreatic stimulation and impaired temporal coordination between gastric emptying and pancreatic protease delivery. Concurrently, bile acid-mediated gastric dysfunction and rapid gastric emptying may further impair acid–pepsin preprocessing of muscle tissue before entry into the small intestine. Loss of collagen-rich structural prey components may further limit mechanical and enzymatic disintegration of muscle fibers within the stomach.

Downstream, dysregulated intestinal motility may shorten transit time and reduce effective mixing of chyme with pancreatic and brush-border enzymes. Together, these factors could permit large muscle fragments to traverse the gastrointestinal tract with incomplete digestion, resulting in visible muscle fibers in feces. Protein maldigestion in these cases could therefore reflect functional failure of gastric–pancreatic coordination and substrate processing rather than primary enzyme deficiency.

Importantly, functional protein maldigestion and fat maldigestion likely arise in parallel from the same upstream disturbances. Both contribute to poor body condition, nutritional instability, and chronic gastrointestinal dysfunction while simultaneously amplifying bile acid dysregulation and epithelial stress.

## 6. Pharmacological Insights from Differential Therapeutic Responses

Patterns of therapeutic response observed in captive cheetahs with chronic gastritis and enteropathy may provide important inferential insight into disease pathogenesis. Considered collectively, the variable efficacy of commonly employed treatments is difficult to reconcile fully with models based primarily on infection or primary immune dysregulation but is broadly consistent with a bile acid-driven disorder arising from disrupted digestive coordination.

### 6.1. Antimicrobial Therapy and Secondary Colonization

Antimicrobial therapy directed at *Helicobacter* spp. has been widely employed in cheetahs with lymphoplasmacytic gastritis. While transient histological or clinical improvement may occur, relapse following cessation of therapy is common and durable resolution is rare [5]. This response pattern is difficult to reconcile with a purely infectious primary cause but may be more readily explained if *Helicobacter* organisms function primarily as secondary colonizers within an already injured gastric environment. Reduction in bacterial load may temporarily decrease inflammation without addressing the underlying chemical injury sustaining disease.

Interpretation of treatment response is further complicated by the fact that many clinical cases are managed empirically or diagnosed primarily on histopathology without bacterial culture or antimicrobial susceptibility testing. In this context, variable therapeutic response does not necessarily exclude a contributory role for *Helicobacter* spp. but may suggest that bacterial colonization alone is insufficient to explain the overall disease process.

### 6.2. Limited Efficacy of Glucocorticoid Therapy

If lymphoplasmacytic gastritis were primarily immune-mediated, suppression of lymphocyte activity would be expected to produce predictable and sustained clinical improvement. In practice, however, both systemic glucocorticoids and locally acting agents such as budesonide frequently provide limited or inconsistent benefit in affected cheetahs. Where improvement occurs, relapse following dose reduction or withdrawal is common.

Mechanistically, this lack of response is informative. Glucocorticoids suppress immune effector pathways but do not neutralize bile acids, restore digestive coordination, or protect epithelial membranes from chemical injury. In addition, glucocorticoid therapy may impair epithelial barrier integrity and mucosal repair mechanisms, potentially exacerbating instability within an already dysregulated digestive system [24].

### 6.3. Efficacy of Sulfasalazine in Non-Colitic Disease

By contrast, sulfasalazine frequently appears to produce clinical improvement in cheetahs with chronic gastritis and enteropathy, even in the absence of overt colonic disease. Although sulfasalazine is traditionally regarded as a treatment for inflammatory colitis, its observed efficacy in these cases may be more readily explained within the context of chemically driven mucosal injury.

Sulfasalazine and its active moiety, 5-aminosalicylic acid, inhibit NF-κB-mediated inflammatory signaling and stabilize epithelial barrier function [23,25]. These effects could partially counter bile acid-mediated epithelial injury and secondary immune activation without relying on deep immunosuppression. Importantly, sulfasalazine would not be expected to correct bile acid mislocalization or impaired digestive coordination directly. Rather, it may improve mucosal tolerance to ongoing epithelial stress, thereby enhancing clinical stability while upstream physiological disturbances persist.

### 6.4. Differential Response to Pancreatic Enzyme Supplementation

Patterns of response to pancreatic enzyme supplementation may provide further mechanistic insight. Supplementation with proteolytic enzymes alone, including plant-derived proteases, frequently fails to improve fecal quality or gastrointestinal stability. In contrast, preparations containing pancreatic lipase often appear to produce more substantial improvement in stool consistency and nutrient utilization.

This response pattern suggests that fat digestion may represent a particularly important limiting step in many affected cheetahs. Importantly, failure of protease-only supplementation does not exclude concurrent protein maldigestion. Rather, it may reflect impaired gastric preprocessing, suboptimal pancreatic stimulation, and reduced temporal coordination between enzyme delivery and substrate availability. The apparent efficacy of lipase-containing preparations, despite the lack of evidence for primary pancreatic exocrine insufficiency, supports the possibility of functionally impaired digestive coordination rather than true enzyme deficiency.

### 6.5. Therapeutic Modulation of Bile Acids

Agents capable of modifying bile acid availability or composition may provide additional support for a bile acid-centered mechanism. Bile acid sequestrants such as cholestyramine may improve diarrheal signs by reducing colonic exposure to irritant bile acids, while hydrophilic bile acids, including ursodeoxycholic acid and tauroursodeoxycholic acid, may reduce cytotoxicity and stabilize bile acid-mediated signaling pathways [16,18,26]. These agents are unlikely to be curative when used in isolation but may reduce inflammatory reactivity and relapse frequency when integrated into a broader, physiology-based management strategy.

### 6.6. Integrating Therapeutic Response into Pathogenesis

Considered individually, therapeutic responses in captive cheetahs may appear inconsistent. Viewed collectively, however, a broader pattern emerges. Interventions aimed solely at suppressing immune activity often provide incomplete benefit, whereas therapies that improve digestive coordination, enhance fat digestion, reduce bile acid toxicity, or stabilize epithelial inflammatory signaling seem to produce more consistent clinical improvement. Furthermore, feeding interventions that restore feast–fast physiology frequently enhance or exceed pharmacological effects.

These response patterns do not prove the present hypothesis but provide inferential support for a model in which chronic gastritis and enteropathy in captive cheetahs are sustained primarily by ongoing bile acid-mediated epithelial injury arising from disrupted feeding ecology and impaired digestive coordination, rather than by primary immune or infectious disease.

## 7. Implications for Clinical Management and a Physiology-Based Treatment Framework

Reframing lymphoplasmacytic gastritis in captive cheetahs as a consequence of disrupted feeding ecology, functional fat maldigestion, and bile acid mislocalization has important implications for clinical management. Rather than representing separate or coincidental disorders, gastritis, maldigestion, and enteropathy may be better interpreted as interconnected manifestations of a shared upstream physiological disturbance. Effective management may therefore depend less on isolated suppression of downstream inflammation and more on correction of underlying digestive instability.

### 7.1. Feeding Structure as a Primary Therapeutic Consideration

One of the most important implications of this hypothesis is that feeding structure should not be regarded as a neutral husbandry variable but as an important determinant of gastrointestinal health. Restoration of feeding patterns that more closely approximate natural feast–fast ecology may support coordinated interdigestive motility, bile acid recycling, and epithelial recovery. Consolidation of food intake into fewer, larger meals separated by meaningful fasting intervals may therefore represent an important management consideration where practical constraints allow.

Clinical observations further suggest that even small quantities of food provided on intended fasting days can destabilize gastrointestinal function, possibly by interrupting bile acid cycling and digestive coordination without delivering the physiological signals associated with a true feeding event. These observations have direct relevance for training, enrichment, and medical administration practices in captive cheetahs.

### 7.2. Diet Composition and Carcass Structure

Beyond feeding frequency, dietary structure appears central to digestive stability. Diets dominated by lean skeletal muscle differ substantially from whole-prey diets in both structural complexity and connective tissue content [13,14]. Inclusion of collagen-rich tissues and structurally complex carcass components may promote more physiologically appropriate gastric emptying patterns, digestive kinetics, and temporal coordination of nutrient delivery.

Within this framework, collagen-rich tissues are interpreted not simply as protein sources but as functional structural components of the diet that may influence digestive coordination and gastrointestinal stability. Their relative absence from many captive diets could contribute to both functional fat maldigestion and increased susceptibility to bile acid-mediated epithelial stress.

### 7.3. Targeted Support of Fat Digestion

Where clinical signs or fecal characteristics suggest maldigestion, pancreatic enzyme supplementation containing lipase may be beneficial, even in the absence of evidence for primary pancreatic exocrine insufficiency. The observed efficacy of lipase-containing preparations supports the concept of functional enzyme inefficacy driven by disrupted coordination rather than enzyme deficiency.

Within the proposed framework, enzyme supplementation should be regarded primarily as supportive therapy rather than definitive treatment. Durability of clinical response is likely to depend on concurrent correction of feeding structure and diet composition.

### 7.4. Modulation of Bile Acid Toxicity and Signaling

Pharmacological modulation of bile acid exposure and composition may provide additional benefit in selected cases. Bile acid sequestrants such as cholestyramine may reduce colonic exposure to irritant bile acids, while hydrophilic bile acids such as ursodeoxycholic acid or tauroursodeoxycholic acid may reduce cytotoxicity and stabilize bile acid-mediated signaling pathways [16,18,26]. Although unlikely to be curative when used in isolation, these interventions may reduce inflammatory reactivity and relapse frequency when incorporated into a broader physiology-based management strategy.

### 7.5. Reconsidering the Role of Anti-Inflammatory Therapy

Anti-inflammatory therapies should be interpreted within the context of secondary immune activation rather than presumed primary immune dysregulation. The apparent efficacy of sulfasalazine, even in the absence of overt colitis, is consistent with a role in modulating chemically driven mucosal inflammation and epithelial stress. By contrast, the inconsistent response to glucocorticoids suggests that immunosuppression alone may be insufficient when the underlying physiological disturbances persist.

These distinctions have practical importance. Escalation of steroid therapy in refractory cases may expose animals to unnecessary risk without addressing disease drivers, whereas therapies that stabilize epithelial integrity and inflammatory signaling may offer greater benefit with lower systemic impact.

### 7.6. Integrating Management Strategies

Taken together, these considerations support a physiology-based management framework in which priority should be given to restoring appropriate feeding ecology and dietary structure, followed by targeted support of fat digestion and bile acid handling, and finally by modulation of secondary inflammation where required. Such an approach does not preclude individualized treatment but may provide a more coherent biological rationale for clinical decision-making grounded in cheetah-specific digestive physiology.

Importantly, this framework does not suggest that all affected cheetahs will respond identically or that any single intervention is likely to be universally effective. Rather, it proposes that disruption of digestive coordination and bile acid physiology may represent common upstream factors contributing to the development and persistence of gastrointestinal disease in susceptible individuals. The relative contribution of feeding frequency, diet structure, bile acid dysregulation, microbial changes, and secondary inflammation likely varies between animals and over time.

Within this context, therapeutic strategies aimed solely at suppressing inflammation may provide incomplete or temporary benefit if the underlying physiological disturbances remain unresolved. Conversely, management approaches that improve digestive coordination and restore more physiologically appropriate feeding patterns may reduce the downstream inflammatory burden while simultaneously improving digestive stability and nutrient utilization.

### 7.7. Implications for Future Research

The hypothesis presented here generates several testable predictions. Restoration of feast–fast feeding patterns and increased dietary carcass complexity should improve markers of bile acid regulation and digestive stability. Alterations in bile acid composition and signaling pathways should correlate with disease severity and therapeutic response. Interventions that stabilize bile acid physiology should reduce relapse following withdrawal of anti-inflammatory therapy.

Several important components of this proposed mechanism remain incompletely characterized in cheetahs. Direct studies evaluating gastric emptying dynamics, bile acid composition, enterohepatic cycling, fasting-associated motility patterns, and pancreatic-biliary coordination in healthy and affected animals are currently lacking. Likewise, the effects of feeding frequency and carcass structure on digestive physiology in captive cheetahs remain poorly defined.

Prospective studies integrating feeding interventions, bile acid profiling, metabolomics, gastric motility assessment, fecal analysis, and clinical outcomes are needed to evaluate these predictions. Longitudinal studies would be particularly valuable in determining whether alterations in digestive coordination and bile acid physiology precede the development of overt gastritis or arise secondarily during disease progression.

An additional physiological factor that may influence disease progression in affected cheetahs is disturbance of systemic acid–base and electrolyte balance. Gastric acid secretion is physiologically coupled to a transient postprandial rise in systemic bicarbonate, the so-called alkaline tide, a phenomenon documented in several mammalian species including dogs [15,27]. In animals fed infrequently, this transient alkalinization is normally followed by a prolonged interdigestive phase during which acid–base equilibrium is restored and the migrating motor complex re-establishes coordinated gastrointestinal motility. However, frequent feeding may produce repeated or prolonged alkaline tide events while simultaneously suppressing the interdigestive phase and its associated motility cycles, maintaining the gastrointestinal tract in a semi-postprandial physiological state.

In cheetahs experiencing chronic regurgitation or selective loss of gastric hydrochloric acid, this process may predispose to hypochloremic metabolic alkalosis and secondary hypokalemia. Conversely, in individuals where bile acid-associated enteropathy and diarrhea predominate, loss of bicarbonate-rich intestinal fluid may instead lead to hyperchloremic metabolic acidosis. Both hypokalemia and chronic acid–base disturbance can impair smooth muscle excitability and gastrointestinal contractility, potentially exacerbating gastric stasis, duodenogastric reflux, and digestive coordination failure. Characterizing the acid–base and electrolyte profiles of affected cheetahs may therefore provide further insight into inter-individual variation in clinical presentation and may represent an additional avenue for therapeutic intervention.

More broadly, the present hypothesis may also have relevance beyond the cheetah. Other obligate carnivores maintained in captivity are commonly exposed to feeding patterns and diet structures that differ substantially from those encountered under natural conditions. Comparative investigation across species may therefore help determine whether disruption of feeding ecology and bile acid physiology contributes more broadly to gastrointestinal disease in captive carnivores.

## 8. Conclusions

Lymphoplasmacytic gastritis remains one of the most pervasive and clinically important disorders affecting captive cheetahs, yet its underlying pathogenesis has remained unresolved for decades. The hypothesis advanced here proposes that this condition is best understood not as a primary infectious or immune-mediated disease, but as a histological reaction pattern arising from sustained physiological mismatch between natural feeding ecology and management practices in captivity.

By integrating concepts from feeding ecology, diet structure, digestive kinetics, bile acid physiology, and differential therapeutic responses, this review provides a coherent mechanistic framework linking disrupted feast–fast cycles and functional fat maldigestion to bile acid-mediated chemical injury of the gastric mucosa and secondary immune activation. Within this model, the frequent co-occurrence of gastritis and maldigestion is no longer paradoxical but represents parallel manifestations of a shared upstream disturbance.

Recognition of lymphoplasmacytic gastritis as a bile acid-driven gastroenteropathy has important implications for both interpretation of pathology and clinical management. It shifts emphasis away from isolated suppression of inflammation and toward restoration of feeding structure, dietary composition, and digestive coordination that more closely align with cheetah-specific physiology. While further prospective work is required to test specific predictions of this model, the framework presented here offers a biologically plausible and unifying explanation for a long-standing disease of captive cheetahs. If supported by future studies, this model may have broader implications for understanding gastrointestinal disease in other obligate carnivores maintained under captive conditions.

## Data Availability

The original contributions presented in this study are included in the article. Further inquiries can be directed to the corresponding author.
